# Impact of modified psychomotor therapy on self-efficacy in community-dwelling individuals with schizophrenia receiving rehabilitation

**DOI:** 10.3389/fpsyt.2025.1721293

**Published:** 2025-12-16

**Authors:** Huaiming Zhang, Chao Chen, Yijun Chen, Jia Hu, Jiakai Han, Wenxuan Ji, Shasha Wang

**Affiliations:** 1 Mental Health Center Affiliated to Shanghai University of Medicine & Health Sciences, Shanghai, China; 2 Shanghai Yangpu Mental Health Center, Shanghai, China; 3 Tangzhen Community Health Service Center, Shanghai, China; 4 College of Rehabilitation Sciences, Shanghai University of Medicine and Health Sciences, Shanghai, China

**Keywords:** community rehabilitation period, complementary intervention, modify psychomotor therapy, schizophrenia, self-efficacy

## Abstract

**Purpose:**

To investigate the rehabilitative effects of modified psychomotor therapy (mPMT), which incorporates traditional Chinese healthcare exercises, on the self-efficacy, psychiatric symptoms, quality of life, and social functioning of individuals with schizophrenia during the community rehabilitation period.

**Methods:**

A total of 96 individuals with schizophrenia, who were being managed during community rehabilitation period at Yangpu District, were randomly and equally divided, using the simple random number method, into two groups: the intervention group (mPMT) and the control group (Control). The control group received routine community mental health services, including psychiatric medication, follow-up visits from the community family doctor teams, health education and routine community rehabilitation services, whereas the mPMT group received psychomotor therapy alongside these services above for a period of six months. The self-efficacy of individuals with schizophrenia was primarily assessed using the General Self-Efficacy Scale (GSES). Psychiatric symptoms, social disability and quality of life were respectively assessed using the Positive and Negative Syndrome (PANSS), the Social Disability Screening Schedule (SDSS) and the Schizophrenia Quality of Life Scale (SQLS).

**Results:**

After six months, the within-group GSES scores were significantly higher in both the control and mPMT groups compared to the baseline scores (*p* < 0.0001), while the SQLS and SDSS scores were significantly lower (*p* < 0.001). After 6 months of intervention, PANSS, SQLS and SDSS scores were significantly lower in the mPMT group than in the control group (*p* < 0.001).

**Conclusion:**

mPMT serves as an effective complementary intervention to alleviate psychiatric symptoms, enhance self-efficacy, and improve the quality of life for individuals with schizophrenia in community rehabilitation.

Schizophrenia is a psychiatric disorder characterized by cognitive dysfunction, which is a core symptom, as well as positive and negative symptoms (1). It is marked by fundamental changes in personality, fragmented thoughts, emotions, and actions, and a loss of coordination with the surrounding environment. These disturbances not only impair cognitive abilities but also diminish social functioning and self-efficacy—both of which are closely linked to patients’ long-term prognosis (2). According to the World Health Organization’s world mental health report 2022, nearly one billion people around the world suffer from a mental illness, which equates to one in eight people in the world experiencing mental health challenges. Epidemiological data indicate high relapse rates among individuals with psychotic disorders: 53.7 % relapse within two years and 81.9 % within five year (3). Recovery, especially within community settings, remains a formidable challenge, and China’s community-based mental health rehabilitation infrastructure is still underdeveloped, limiting many patients’ opportunities for normal family life and social reintegration (4). Therefore, the pressing issue is how to effectively assist patients with mental illness, who are permanent residents of the community, in enhancing their cognitive abilities and achieving improved social functioning and self-efficacy necessary for better daily living.

Self-efficacy refers to an individual’s belief in his or her capability to perform specific behaviors. It is a pivotal determinant of daily functioning, social participation, and overall quality of life. While antipsychotic medication is essential for preventing relapse, long-term pharmacotherapy can adversely affect quality of life and does not fully restore social functioning or self-efficacy (5). Poor medication adherence and patients’ reluctance to continue oral treatment after symptom stabilization further contribute to relapse (6). Moreover, medication alone has limited impact on negative symptoms, quality of life, and social functioning (7, 8).

Research demonstrates that regular physical exercise promotes neural connectivity, thereby enhancing cognition and memory (9). Exercise also mitigates some of the adverse effects of antipsychotic drugs. Controlled physical activity has been shown to improve psychiatric symptoms, increase motivation for engagement, and encourage active participation in social activities among schizophrenia patients (10, 11). Ho et al. conducted their study in Hong Kong, China—a region combining Eastern cultural values and a mature Western-style mental health service system. The cultural context may have increased participants’ familiarity and willingness to engage in Tai Chi (a traditional Chinese practice), while the community setting reduced hospital-related barriers to consistent participation (12).

In addition to exercise, community-based therapies—such as cognitive-behavioral therapy, skills training, cognitive remediation, peer-support groups, and medication-adherence interventions—help patients cope with difficulties and facilitate societal reintegration (13–16). Yet these approaches are typically delivered in isolation, lacking a comprehensive mind-body integration. Therefore, a rehabilitation method integrating psychotherapy and exercise is needed.

Psychomotor Therapy (PMT) is a multidisciplinary, mind-body treatment that incorporates mental-state analysis, philosophy, neurology, neurophysiology, and cognitive neuropsychology (17). It aims to synchronize physical movement with mental activity, fostering connections between patients, others, and the environment. By viewing physical, mental, and social functions as an integrated whole, PMT employs techniques such as body-perception training to energize both body and mind for therapeutic and rehabilitative purposes (3). Haeyen’s systematic review recommended PMT for personality-disordered individuals, noting improvements in emotional regulation, stress management, and social functioning (18). Nonetheless, research on PMT’s impact on community psychiatric rehabilitation—particularly on self-efficacy—is scarce.

Therefore, the present study aims to utilize the mPMT, which incorporates traditional Chinese health-preserving exercises, to provide a 6-month intervention for individuals with schizophrenia during their community rehabilitation period. The goal is to assess their self-efficacy, negative and positive psychiatric symptoms, and quality of life, among other factors. The present study assumed that mPMT would be effective in improving self-efficacy and quality of life for individuals with schizophrenia during community rehabilitation period.

## 1 Methods

### 1.1 Participants

The study sample was drawn from patients who were currently residing in the Yangpu district during community rehabilitation period. Both patients and their guardians were fully informed before signing an informed consent form. The trial was approved by the ethics committee of Yangpu District Mental Health Center, Shanghai (Approval No. 2021-011).

The sample size was calculated using G*Power software. According to the results of the literature review, an increase of 3 in self-efficacy was categorized as effective. The mPMT group’s effectiveness rate was categorized as ρ1 = 0.75 and the control group’s as ρ2 = 0.40. The confidence level was categorized as α = 0.05, the certainty level as 1-β = 0.90 and the tolerance level as δ = 0.1. This resulted in a bilateral Z1-α/2 = 1.96 and Z1-β = 1.28. The required sample size was calculated to be 40 cases for each group. Based on an expected dropout rate of 20%, the required sample size is 48 cases per group for a total of 96 cases. The 96 patients were randomly and equally divided into control and mPMT groups using the random number table approach. Participants were unaware of their assigned number until the baseline assessment had been completed. The physicians involved in the assessment were trained in homogenization and were blinded to the trial grouping information to ensure an accurate assessment. A group of community workers and physicians contacted the subjects closely by telephone and home visits to improve their compliance.

Inclusion criteria: meet the diagnostic criteria of schizophrenia of *“International Classification of Diseases ICD-10”*; discharge time < 2 years or disease duration <15 years; age 18~60 years old, junior high school or above, male or female; stable condition, able to communicate normally, and able to cooperate with the completion of the various experimental evaluations; did not undergo any other rehabilitation training in the 6 months prior to the entry into this study; and did not perform physical exercise regularly in the normal course of the day.

Exclusion criteria: those who do not meet the diagnostic criteria of schizophrenia of *“International Classification of Diseases (ICD-10)”*; those who do not meet the above inclusion criteria; those with severe skeletal and muscular system, neurological disease; those with drug addiction or abuse; those with psychotic episodes; those with critical conditions (e.g. severe cardiac insufficiency, serious infections, and tumors); those who refuse to be followed up, and those who are not able to independently complete all the above evaluations and rehabilitation training.

### 1.2 Intervention method

Both the intervention and control groups received routine community mental health services, including psychiatric medication, follow-up visits by community family doctor teams, health education and routine community rehabilitation services. The intervention group received mPMT alongside the above routine community mental health services for a total of 6 months (25 weeks). Each mPMT session lasted 90 minutes, consisting of three 30-minute modules: relaxed perception training (first 30 mins), Thematic Course (middle 30 mins), and relaxed perception training (last 30 mins). For the epidemic prevention-related training, there will be 3 sessions per week from Week 1 to Week 8 (combining online and offline methods) to ensure an 80% participation rate; 2 sessions in Week 9; and 1 session per week from Week 10 to Week 25 (also combining online and offline methods). For patients who cannot attend offline training, online training will be arranged as an alternative, and the overall training participation rate shall be no less than 75%. The detailed weekly curriculum is presented in **Supplementary 1**.

### 1.3 Assessment methods

#### 1.3.1 General self-efficacy scale

The GSES consists of 10 items designed to assess an individual’s level of confidence in the face of frustration or adversity on a 4-point Likert scale (“not at all true”, “somewhat true”, “mostly true” or “completely true”) (19). The scale is scored as follows: 1 for “not at all correct,” 2 for “somewhat correct,” 3 for “mostly correct,” and 4 for “completely correct.” The GSES is a unidimensional scale, and only the total scale score is calculated. This total is obtained by summing the scores of all 10 items and then dividing by 10.

#### 1.3.2 Positive and negative syndrome

The PANSS is used to measure the severity of symptoms in schizophrenia patients and to determine whether psychopathic symptoms are present (20). Psychiatrists are trained in the application of measurement and implement a comprehensive evaluation of the adult, taking into account information from the clinical examinations, family members, and other aspects of the patient’s life in order to ensure that the results are accurate and trustworthy. Typically, the evaluation consists of all patient information over a one-week period.

#### 1.3.3 Social disability screening schedule

The SDSS is utilized to evaluate the extent of social disability among psychiatric patients (21). It comprises 10 items, each scored on a scale from 0 to 2: (0) signifies no significant functional impairment, (1) indicates significant functional impairment, and (2) denotes severe functional impairment. During the evaluation process, the evaluator needs to be professionally trained and spends 5–8 minutes on questioning. For some special cases, there may perhaps be exclusions, such as assessments 2 and 3 for unmarried people, which can be recorded in (9) and will not be counted in the overall score. According to the latest regulations, the assessment is limited to the last month. Each assessment requires 5–10 minutes. The SDSS assessment indicators encompass the total score and individual item scores. Based on the results of the national epidemiological survey on mental illness across twelve provinces, an individual scoring ≥ 2 is identified as having a social functioning disability. This threshold is also utilized in the China Disability Prevalence Survey to determine mental disability (22).

#### 1.3.4 The schizophrenia quality of life scale

The SQLS is a scale developed in 1999 by British psychiatrists Greg Wilkinson and Dlane Wild to measure the quality of life of people with mental disorders so that they can better understand how they are living. The scale consists of 30 entries: 15 psychosocial subscales and 7 motivational/spiritual subscales reflecting emotional expression and interpersonal interactions as well as motivational and spiritual components, respectively. In addition, there are eight symptom/side effect subscales, which reflect the side effects of medication. Responses were scored on a five-point scale ranging from 0-4. The raw scores of this questionnaire were converted to a final standardized score of 0-100, where lower scores indicate a better subjective quality of life.

### 1.4 Statistical analysis

SPSS 22.0 statistical software was used for analysis. Continuous variables were tested for normality and homoscedasticity by Wilk’s test. Information on measures conforming to normal distribution was expressed as mean ± standard deviation (x̄± s), inter- and intra-group comparisons were made by independent samples T-tests, and non-normal distributions were expressed as median (P25, P75), with non-normal distributions tested using the Mann-Whitney non-parametric test. Categorical data were tested using the chi-square test or Fisher’s exact test. Statistical results *p* < 0.05 indicates statistically significant differences.

## 2 Results

### 2.1 Baseline characteristics

Comparing the general information of the intervention and control groups, there were no significant differences between the two groups in terms of gender, education, medication use, marital status, family history, form of onset of the disease, proportion of psychiatric symptoms, age and duration of the disease. (**Table 1**).

**Table 1:** Baseline general population characteristics of the two groups.

		Ctrl (n=48)	Intv (n=48)	Z/t/x^2^	*p*
Population characteristic					
Sex (n,%)	Male	22 (45.8)	23 (47.9)	0.042	0.838
	Female	26 (54.2)	25 (52.1)		
Age (Mean ± SD)		43.6 ± 5.7	44.7 ± 6.0	0.901	0.367
Education (n,%)	Junior High	6 (12.5)	8 (16.7)	1.151	0.765
	Senior High	17 (35.4)	18 (37.5)		
	Associate	13 (27.1)	14 (29.2)		
	Bachelor+	12 (25.0)	8 (16.7)		
Marital (n,%)	Single	26 (54.2)	32 (66.7)	1.806	0.386
	Married	17 (35.4)	11 (22.9)		
	Divorced	5 (10.4)	5 (10.4)		
Family Hx (n,%)	N	45 (93.8)	44 (91.7)	0.000	1.000
	Y	3 (6.2)	4 (8.3)		
Clinical characteristics					
Duration (Mean ± SD)		2.0(2.0,4.0)	4.0(2.0,7.0)	-2.006	0.045*
Medication (n,%)	Monotherapy	8 (16.7)	9 (18.8)	0.072	0.789
	Combination therapy	40 (83.3)	39 (81.2)		
Onset (n,%)	Acute	7 (14.6)	6 (12.5)	0.186	0.911
	Subacute	16 (33.3)	15 (31.2)		
	Chronic	25 (52.1)	27 (56.2)		
Symptom Types (n,%)	0	5 (10.4)	9 (18.8)	6.728	0.081
	1	24 (50.0)	12 (25.0)		
	2	10 (20.8)	16 (33.3)		
	3+	9 (18.8)	11 (22.9)		
Support Group (n,%)	N	13 (27.1)	14 (29.2)	0.052	0.821
	Y	35 (72.9)	34 (70.8)		

**Figure 1:**
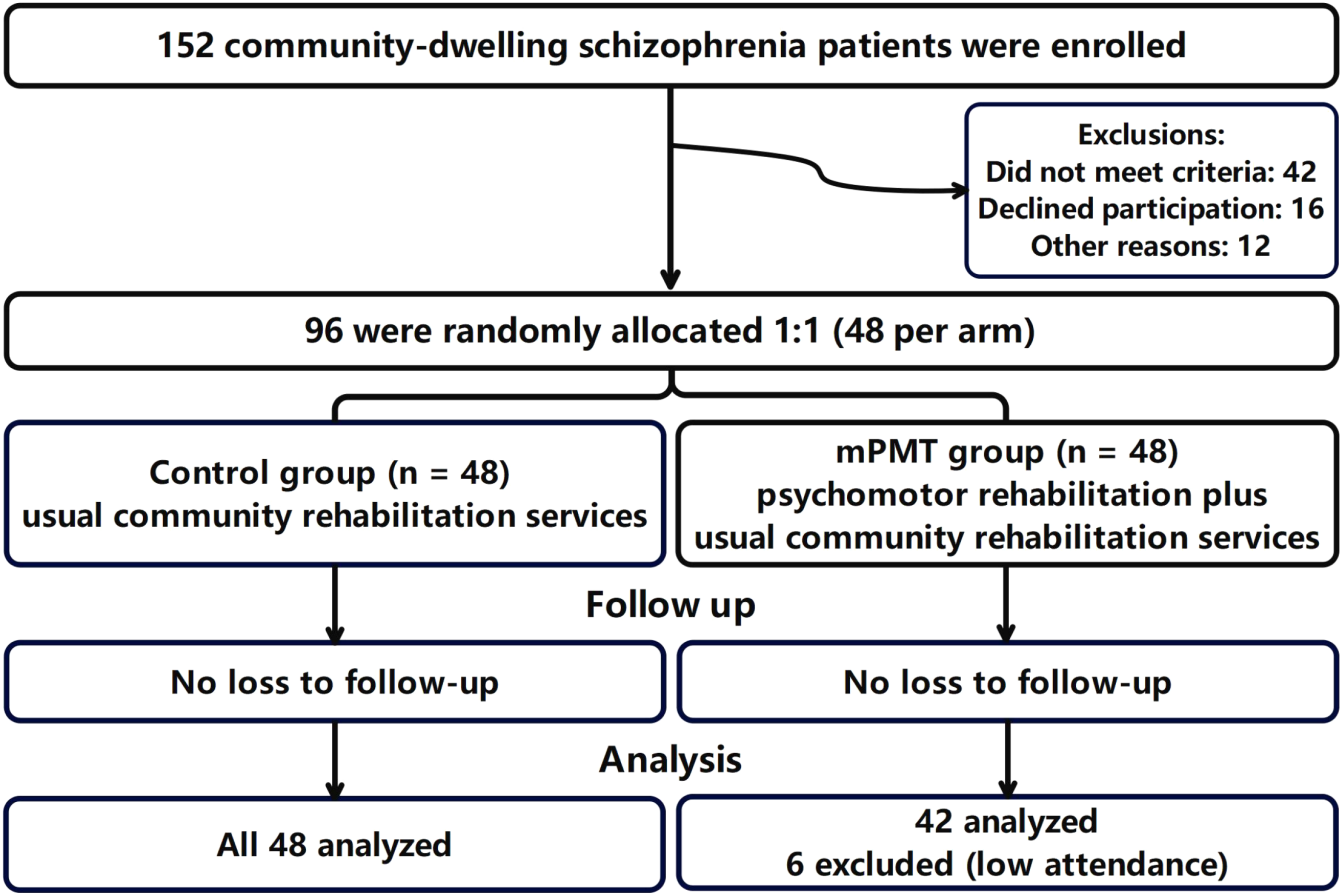
Flow chart.

Over the course of the 6-month intervention, there was a 12.5% dropout rate in the mPMT group, with 6 patients interrupting their training during psychomotor processes (**Figure 1**).

### 2.2 Main outcome indicators

#### 2.2.1 Effects of the mPMT intervention on self-efficacy

**Figure 2:**
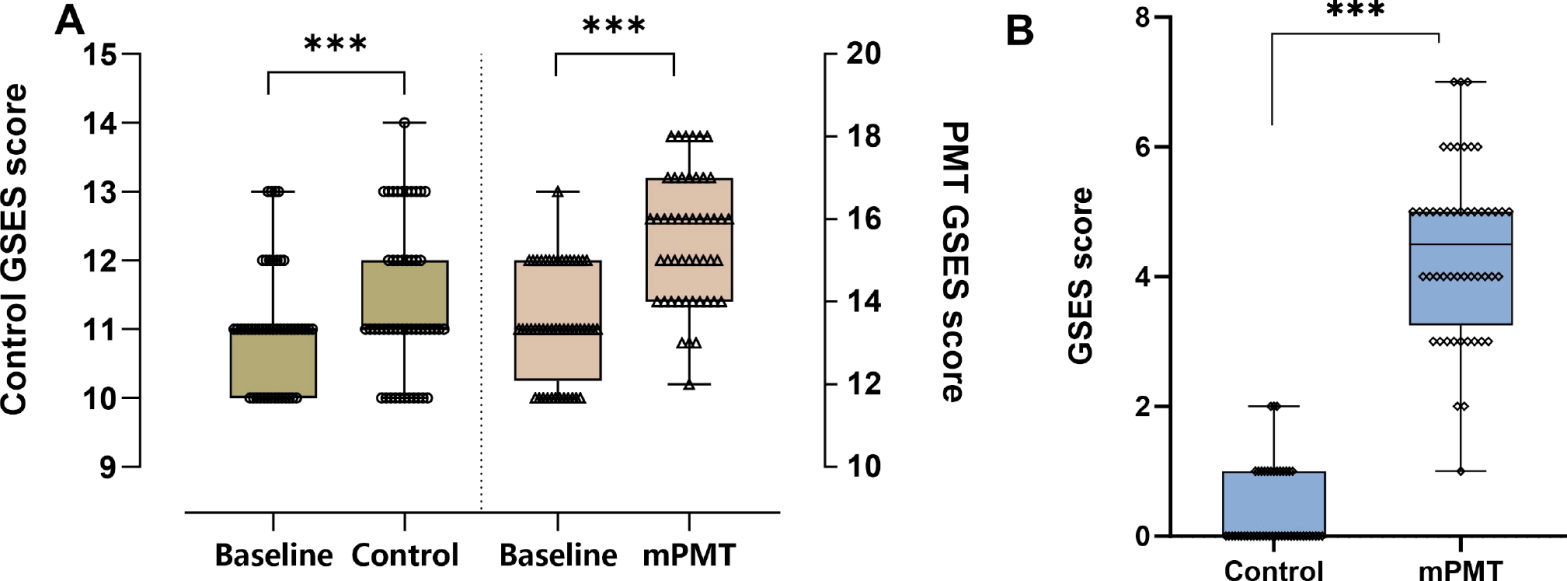
Effect of mPMT intervention on GSES. **(A)** Intragroup comparison between the intervention and control groups, with the left y-axis showing GSES scores in the control group and the right y-axis showing GSES scores in the mPMT group; **(B)** Intergroup comparison between the intervention and control groups. ***p < 0.0001.

Self-efficacy directly affects behavioral choices, motivation, and emotional responses. GSES scale scores were significantly higher in the mPMT group compared to baseline after 6 months of mPMT intervention (z = 6.071, *p* < 0.0001) and in the control group compared to baseline after routine mental health services (z = 3.755, *p* < 0.0001). But the difference in GSES scale scores between the two groups was significantly higher in the mPMT group -4.42(95% CI, -4.8‐‐4.03) than in the control group -0.4 (95% CI, -0.57‐‐0.22)(Cohen’s d = 3.96; z = 8.553, *p* < 0.0001) (**Figure 2**). This suggests that mPMT therapy leads to greater improvements in patients’ self-efficacy.

### 2.3 Secondary outcome indicators

#### 2.3.1 Effects of mPMT intervention on PANSS, SDSS, SQLS

**Figure 3:**
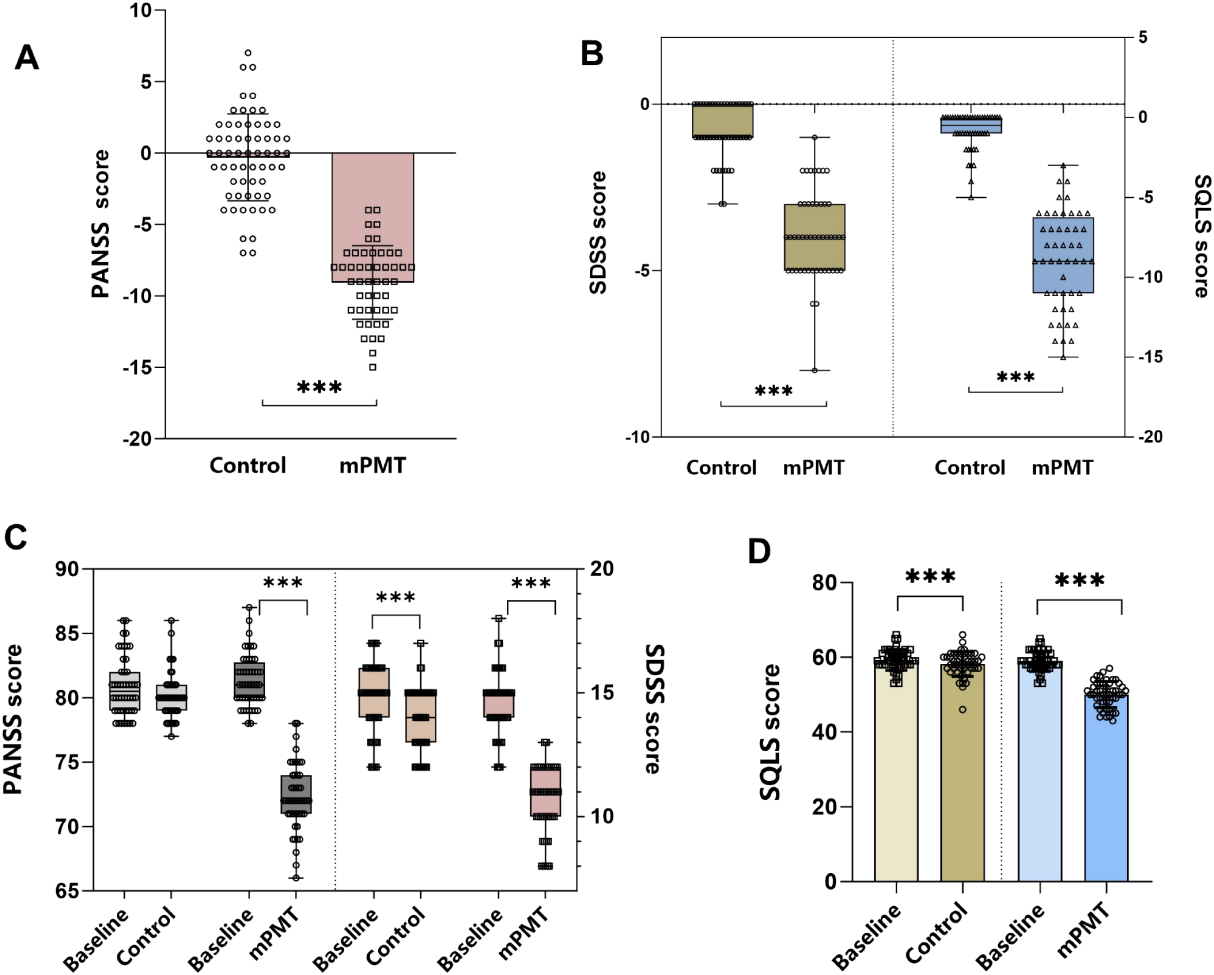
Secondary outcome indicators. **(A)** Comparison of the difference in PANSS scores between the control and mPMT groups; **(B)** Comparison of the difference in SDSS and SQLS scores between the control and mPMT groups; **(C)** Comparison of the PANSS and SDSS of the control and mPMT groups with the baseline data at 6 months post-intervention; **(D)** Comparison of the SQLS of the control and mPMT groups with the baseline data at 6 months post-intervention. ***p < 0.0001.

The PANSS was used to evaluate the severity of symptoms in different types of schizophrenia, and through the 6-month intervention, the total scores of the mPMT group were all significantly lower compared to baseline (z = -6.043, *p* < 0.0001) (**Figure 3C**), but there was no significant difference in the control group (z = -1.715, *p* = 0.086). Moreover, the mPMT group 9.06(95% CI,8.32-9.81) was more effective in reducing the degree of symptoms compared to the control group 0.77(95% CI, -0.08-1.62) (Cohen’s d = 3.15; z = -14.746, *p* < 0.0001) (**Figure 3A**).

The SDSS scores were primarily used with psychiatric patients living in the community to assess the degree of impairment in social functioning in psychiatric patients. After 6 months of intervention, both the control group and the mPMT group showed a significant reduction from baseline (z = -4.813, *p* < 0.0001; z = -6.077, *p* < 0.0001) (**Figure 3C**). However, comparing the intervention effects between the two groups, it was found that the difference in SDSS scores was significantly lower in the mPMT group 3.94(95% CI, 3.55-4.32) than in the control group 0.81(95% CI, 0.57-1.06) (Cohen’s d = 2.9; z = -8.084, *p* < 0.0001) (**Figure 3B**). Therefore, the mPMT intervention had a more favourable impact on patients’ social functioning.

To assess whether patients’ quality of life improved after the intervention, we assessed the improvement in quality of life using the SQLS scale. Significant improvement in quality of life occurred in both the control and mPMT groups after the 6-month intervention (59.35 ± 2.82 vs. 58.48 ± 2.84; t = -5.145, *p* < 0.0001) VS (58.83 ± 2.64 vs. 49.94 ± 3.52; t = -20.318, *p* < 0.0001) (**Figure 3D**). Despite the improvement in quality of life in both groups, the mPMT group 8.9(95% CI, 8.02-9.78) remained significantly better than the control group 0.88(95% CI, 0.53-1.22), with a significant reduction in SQLS score (Cohen’s d = 3.52; z = -8.474, *p* < 0.0001) (**Figure 3B**). A significant negative correlation exists between GSES and SQLS (r = -0.287, *p* = 0.049), indicating that higher GSES correlates with better SQLS -where lower scores indicate higher quality of life.

## 3 Discussion

The novelty of mPMT lies in its incorporation of traditional Chinese health exercises, which are rooted in the concept of holistic mind-body unity. It is a specialized, body-mediated, and functionally re-engineered rehabilitation process designed to foster psychological adjustment. After 6 months of mPMT intervention, the self-efficacy and scores of all subscales in the mPMT group were significantly different compared with the pre-intervention period, suggesting that mPMT was able to improve the self-efficacy and quality of life of individuals with schizophrenia in the community rehabilitation period.

Self-efficacy and positive or negative coping styles as interlocking mediators in the relationship between psychological resilience and depression (10). The results of the study showed that compared with the control group, patients in the mPMT group showed significant improvement in their self-efficacy after 6 months of intervention with mPMT, suggesting that mPMT can effectively improve the self-efficacy of individuals with schizophrenia in the community rehabilitation period. The significant improvement in self-efficacy observed in the mPMT group can be effectively interpreted using Albert Bandura’s theory of self-efficacy. This theory posits that an individual’s belief in their capability to execute courses of action is built upon four primary sources: mastery experiences, vicarious experiences, verbal persuasion, and physiological/affective states. The design of mPMT strategically incorporates elements that target these sources. The mPMT program, through its structured progression of exercises such as Tai Chi and Baduanjin, emphasizes motor coordination and relaxation, as well as aerobic exercises, which offer significant benefits in enhancing patients’ physical health, self-confidence, and sense of achievement. They can also motivate patients to participate in physical exercise more actively and improve their adherence to exercise (11). Tai Chi exercise significantly reduces motor disabilities and increases backward digit span and mean cortisol levels in patients (12). Baduanjin improves balance function, motor dual-task performance, and cognitive dual-task performance in individuals with schizophrenia (23). The improvement effect of Baduanjin on logical memory in long-term hospitalized individuals with schizophrenia was also significant (24).

The therapeutic benefits of mPMT extend beyond self-efficacy, exerting positive effects across multiple domains that are best understood within the Bio-psycho-social medical model. A meta-analysis by Vancampfort et al. (25)systematically demonstrated that exercise interventions significantly improve psychological, cognitive, and physical outcomes in people with mental disorders. In line with this, psychomotor rehabilitation builds upon the patient’s self-perception and integrates positive thinking techniques to facilitate cognitive and attitudinal shifts (26, 27). This process encourages a deeper self-understanding, helps patients identify personal strengths and limitations, and ultimately strengthens their self-awareness and belief in their own capabilities. Cognitive self-efficacy in individuals with schizophrenia is a person’s belief in his or her ability to use situational self-regulation, efficacy, and flexibility, as well as interpersonal and intrapersonal coping, and is an important predictor of social functioning (28). Moreover, aerobic exercise has been shown to improve responsiveness to external stimuli, boost cognitive performance, encourage community involvement, and support mental health, all of which contribute to better social functioning (29). From a neurobiological perspective, schizophrenia is associated with dysregulation in neurotransmitter systems, including serotonin and dopamine (30). Appropriate physical activity can modulate the release and availability of these neurotransmitters in the synaptic cleft, leading to improved attentional control and facilitating more effective social communication (31). Furthermore, as negative symptoms diminish, neural connectivity among the prefrontal cortex, striatum, thalamus, and temporal lobe is strengthened, which enhances the brain’s capacity for information processing and emotional regulation (32, 33).

Community-based psychiatric rehabilitation serves as a crucial step in reintegrating home-treated patients into their families and society (34). Studies have shown that rehabilitation activities carried out in specialized centers significantly enhance patients’ medication adherence, daily living skills, and coping abilities. For example, a community-based Tai Chi intervention was found to reduce patients’ scores on the PANSS, decrease the risk of aggressive behavior, and lead to greater improvement in medication adherence one year after the intervention (35). Furthermore, diverse rehabilitation activities have enriched patients’ daily lives, with many valuing the friendships, sense of belonging, and self-worth gained during the process. However, this institution-centric rehabilitation model may, to some extent, slow down patients’ further integration into broader society (36). According to Maslow’s theory of needs, these patients have already met the needs of both physiological and safety levels, so the next goal is the need for belonging and respect. Yet due to constraints in staffing and space, rehabilitation centers are often unable to continuously meet all patients’ recovery needs.

This study has several limitations. First, the samples were geographically restricted to Yangpu District, Shanghai, with a small size (48 participants per group), and the participants were limited to patients with stable conditions, junior high school education or above, and good cooperation ability—failing to cover those with more severe conditions, lower educational levels, or poorer communication skills, which may affect result generalizability. Second, mPMT is a comprehensive intervention involving Taichi, Baduanjin, meditation, and breathing exercises, making it hard to identify the dominant effective component. Third, primary outcome measures such as GSES and SQLS relied on patient self-reports, which are prone to social desirability bias or subjective deviation.

Based on the findings and limitations of this study, several directions for future research are recommended. It is possible that the observed results were inflated by the increased level of care and interaction with medical personnel provided to the mPMT group, as such contact is a recognized beneficial factor in schizophrenia. Conducting comparative effectiveness trials, for instance, directly pitting mPMT against interventions like cognitive behavioral therapy or standard physical exercise, would help to better delineate its specific therapeutic benefits. Furthermore, longitudinal studies with extended follow-up periods are needed to evaluate the long-term sustainability of the improvements in self-efficacy, social functioning, and symptom reduction. Finally, research exploring the differential effects of mPMT across various patient subgroups—such as the elderly, those with significant comorbidities, or individuals at different stages of illness—could help to identify which populations benefit most, thereby facilitating more personalized and effective rehabilitation strategies.

## 4 Conclusion

A comprehensive analysis indicates that a community-managed psychomotor intervention integrating elements of traditional Chinese healthcare exercise can significantly improve self-efficacy, social functioning, and psychotic symptoms in individuals with schizophrenia. These improvements subsequently enhance self-identity and quality of life, supporting the use of this intervention as an adjunctive therapy to pharmacotherapy in community healthcare.

## Data Availability

The raw data supporting the conclusions of this article will be made available by the authors, without undue reservation.
