# Supplementary material for: Impact of modified psychomotor therapy on self-efficacy in community-dwelling individuals with schizophrenia receiving rehabilitation
Source: Front Psychiatry. 2025 Dec 16;16:1721293. doi: 10.3389/fpsyt.2025.1721293 (PMC12748230; doi:10.3389/fpsyt.2025.1721293)
Supplement: Supplementary file 1 [file Table1.docx]

| **Week** | **Sensory Relaxation Training**  **(30 mins)** | **Thematic Course (30 mins)** | **Sensory Relaxation Training**  **(30 mins)** |
| --- | --- | --- | --- |
| Week 1 | Sitting Meditation with Breath Awareness | Massage Ball Stimulation | Sitting Meditation with Breath Awareness |
|  | Body Scanning | Finger Exercises | Body Scanning |
|  | Non-judgmental Awareness | Mirror Imitation Training | Non-judgmental Awareness |
| Week 2 | Progressive Muscle Relaxation | Spatial Motor Perception Training | Progressive Muscle Relaxation |
|  | Sitting Meditation with Breath Awareness | Modified Tai Chi (Form 1) | Sitting Meditation with Breath Awareness |
|  | Body Scanning | Modified Tai Chi (Form 2) | Body Scanning |
| Week 3 | Non-judgmental Awareness | Massage Ball Stimulation | Non-judgmental Awareness |
|  | Progressive Muscle Relaxation | Finger Exercises | Progressive Muscle Relaxation |
|  | Sitting Meditation with Breath Awareness | Mirror Imitation Training | Sitting Meditation with Breath Awareness |
| Week 4 | Body Scanning | Spatial Motor Perception Training | Body Scanning |
|  | Non-judgmental Awareness | Modified Tai Chi (Form 1) | Non-judgmental Awareness |
|  | Progressive Muscle Relaxation | Modified Tai Chi (Form 2) | Progressive Muscle Relaxation |
| Week 5 | Sitting Meditation with Breath Awareness | Error-free Replication Training ("Copy Without Deviation") | Sitting Meditation with Breath Awareness |
|  | Body Scanning | Acupoint Massage for Joints | Body Scanning |
|  | Non-judgmental Awareness | Mirror Imitation Training | Non-judgmental Awareness |
| Week 6 | Progressive Muscle Relaxation | Massage Ball Stimulation | Progressive Muscle Relaxation |
|  | Sitting Meditation with Breath Awareness | Modified Tai Chi (Form 3) | Sitting Meditation with Breath Awareness |
|  | Body Scanning | Modified Tai Chi (Form 4) | Body Scanning |
| Week 7 | Non-judgmental Awareness | Error-free Replication Training ("Copy Without Deviation") | Non-judgmental Awareness |
|  | Progressive Muscle Relaxation | Acupoint Massage for Joints | Progressive Muscle Relaxation |
|  | Sitting Meditation with Breath Awareness | Mirror Imitation Training | Sitting Meditation with Breath Awareness |
| Week 8 | Body Scanning | Massage Ball Stimulation | Body Scanning |
|  | Non-judgmental Awareness | Modified Tai Chi (Form 3) | Non-judgmental Awareness |
|  | Progressive Muscle Relaxation | Modified Tai Chi (Form 4) | Progressive Muscle Relaxation |
| Week 9 | Sitting Meditation with Breath Awareness | Massage Ball Stimulation | Sitting Meditation with Breath Awareness |
|  | Body Scanning | Finger Exercises | Body Scanning |
| Week 10 | Non-judgmental Awareness | Spatial Motor Perception Training | Non-judgmental Awareness |
| Week 11 | Progressive Muscle Relaxation | "Beads Walking Thousands of Miles" (Group Collaboration) | Progressive Muscle Relaxation |
| Week 12 | Sitting Meditation with Breath Awareness | Baduanjin (Form 1) | Sitting Meditation with Breath Awareness |
| Week 13 | Body Scanning | Baduanjin (Form 2) | Body Scanning |
| Week 14 | Non-judgmental Awareness | Massage Ball Stimulation | Non-judgmental Awareness |
| Week 15 | Progressive Muscle Relaxation | Finger Exercises | Progressive Muscle Relaxation |
| Week 16 | Sitting Meditation with Breath Awareness | Spatial Motor Perception Training | Sitting Meditation with Breath Awareness |
| Week 17 | Body Scanning | "Beads Walking Thousands of Miles" (Group Collaboration) | Body Scanning |
| Week 18 | Non-judgmental Awareness | Baduanjin (Form 1) | Non-judgmental Awareness |
| Week 19 | Progressive Muscle Relaxation | Baduanjin (Form 2) | Progressive Muscle Relaxation |
| Week 20 | Sitting Meditation with Breath Awareness | Fun Table Tennis (Pair/Group Interaction) | Sitting Meditation with Breath Awareness |
| Week 21 | Body Scanning | Error-free Replication Training ("Copy Without Deviation") | Body Scanning |
| Week 22 | Non-judgmental Awareness | Acupoint Massage for Joints | Non-judgmental Awareness |
| Week 23 | Progressive Muscle Relaxation | Massage Ball Stimulation | Progressive Muscle Relaxation |
| Week 24 | Sitting Meditation with Breath Awareness | Baduanjin (Form 3) | Sitting Meditation with Breath Awareness |
| Week 25 | Body Scanning | Baduanjin (Form 4) | Body Scanning |
